# Abnormal expression of an *ADAR2* alternative splicing variant in gliomas downregulates adenosine-to-inosine RNA editing

**DOI:** 10.1007/s00701-014-2004-1

**Published:** 2014-02-11

**Authors:** Jun Wei, Zhaohui Li, Chao Du, Bin Qi, Xingli Zhao, Liping Wang, Lirong Bi, Guan Wang, Xuan Zhang, Xiaoyun Su, Yuzhuo Pan, Yu Tian

**Affiliations:** 1 Department of Neurosurgery, China–Japan Union Hospital, Jilin University, Changchun, 130033 China; 2 Department of Neurosurgery, the First Hospital of Jilin University, Changchun, 130021 China; 3 Department of Pathology, China–Japan Union Hospital, Jilin University, Changchun, 130033 China; 4 Department of Pathology, the First Hospital of Jilin University, Changchun, 130033 China; 5 Department of Science and Education, the Second Hospital of Jilin University, 130012 Changchun, China; 6 College of Pharmacy, Jilin University, Changchun, 130021 China; 7 Department of Pharmaceutical Science, School of Pharmacy and Pharmaceutical Science, State University of New York Buffalo, 559 Cooke Hall, Buffalo, NY 14260 USA

**Keywords:** Glioma, ADAR2, Self-editing, ASVs

## Abstract

**Background:**

RNA editing is catalyzed by adenosine deaminases acting on RNA (ADARs). ADAR2 is the main enzyme responsible for recoding editing in humans. Adenosine-to-inosine (A-to-I) editing at the Q/R site is reported to be decreased in gliomas; however, the expression of *ADAR2* mRNA was not greatly affected.

**Methods:**

We determined *ADAR2* mRNA expression in human glioblastoma cell lines and in normal human glial cells by real-time RT-PCR. We also determined *ADAR2* mRNA expression in 44 glioma tissues and normal white matter. After identifying an alternative splicing variant (ASV) of ADAR2 in gliomas, we performed sequencing. We then classified glioblastomas based on the presence (+) or absence (–) of the ASV to determine the correlations between ASV + and malignant features of glioblastomas, such as invasion, peritumoral brain edema, and survival time.

**Results:**

There were no significant differences in *ADAR2* mRNA expression among human glioblastoma cell lines or in gliomas compared with normal white matter (all *p* > 0.05). The ASV, which contained a 47-nucleotide insertion in the *ADAR2* mRNA transcript, was detected in the U251 and BT325 cell lines, and in some glioma tissues. The expression rate of ASV differed among gliomas of different grades. ASV + glioblastomas were more malignant than ASV – glioblastomas.

**Conclusions:**

ADAR2 is a family of enzymes in which ASVs result in differences in enzymatic activity. The *ADAR2* ASV may be correlated with the invasiveness of gliomas. Identification of the mechanistic characterization of *ADAR2* ASV may have future potential for individualized molecular targeted-therapy for glioma.

## Introduction

Gliomas are the most common primary malignant brain tumor in humans. They can be typed as low-grade astrocytomas (LGA), oligodendrogliomas (OG), anaplastic astrocytomas (AA), and glioblastomas multiforme (GBM), among other subtypes. These tumors are often invasive, making surgical resection difficult. Despite the best treatment regimen currently available, which consists of surgery, radiotherapy, and systematic chemotherapy, the prognosis of patients with gliomas is poor. The 5-year survival of patients with GBM is less than 3 % [4, 32].

The widespread RNA–DNA variations found in human brain tissues indicate that RNA sequences are not identical to their corresponding DNA sequences [19]. Most RNA–DNA differences are caused by RNA editing, a process that generates many different mRNAs from the same gene after the post-transcriptional events [2, 3, 8, 23]. The potential impact of RNA editing on the etiology or progression of human diseases has now been realized. Deficient or hyperactive adenosine-to-inosine (A-to-I) RNA editing is associated with several human diseases including epilepsy, malignant brain cancer, amyotrophic lateral sclerosis, immunological disorders, and depression [13, 21, 31]. The analysis of RNA editing in various cancers, including brain, prostate, lung, kidney, bladder, colorectal, and testicular tumors, is expected to provide new diagnostic and prognostic markers [6, 7, 11, 12, 26, 28].

In mammals, RNA editing by site-selective adenosine deamination regulates the key functional properties of neurotransmitter receptors in the central nervous system [2]. Many RNA editing sites have now been reported [3, 8, 9, 21]. Almost all editing of the glutamate receptor (GluR) subunit B (GluR-B) occurs at one position, the Q/R-site, in a process that is essential for normal receptor function [4, 16, 34]. A general decrease in RNA editing activity has been observed in malignant gliomas in the brain. The Q/R site of GluR-B is frequently underedited in malignant gliomas compared with control tissues [5, 22, 32]. Alu sequences within *MED13* transcripts are also underedited in brain tumors [26]. RNA editing is performed by adenosine deaminases acting on RNA (ADARs), of which ADAR2 is the main enzyme responsible for recoding editing in the brain [24, 31]. However, the *ADAR2* mRNA expression levels in gliomas were inconsistent in three previous reports. Furthermore, there was no consistent correlation between editing efficiency and *ADAR2* mRNA expression level in those studies [5, 22, 26, 32]. Therefore, the regulation of the RNA editing activity of *ADAR2* in vivo is still largely unknown.

In this study, we determined *ADAR2* mRNA expression levels in glioma-derived cell lines and in glioma tissues. We have presented evidence for an alternative splicing variant (ASV) of *ADAR2* in gliomas. Furthermore, we explored the correlation between *ADAR2* ASV expression and the invasiveness of GBM. We demonstrate that (1) *ADAR2* mRNA levels were not altered in glioma-derived cell lines or in glioma tissues; (2) self-editing of *ADAR2* pre-mRNA generates an *ADAR2* ASV in glioma-derived cell lines and glioma tissues; and (3) the *ADAR2* ASV may be correlated with the invasiveness of gliomas.

## Materials and methods

### Clinical specimens and cell lines

Glioma tissues and normal white matter tissue samples were obtained from the China–Japan Union Hospital and the First Hospital of Jilin University, Changchun, China between January 2007 and June 2008. Tumor tissue samples were taken from 44 patients with a primary glioma (age range at the time of diagnosis, 17–55 years). None of the patients previously received radiotherapy or chemotherapy. Tumor tissue samples were dissected from both the core and the edge of the tumors. Diagnosis was defined according to the guidelines of the World Health Organization, and was obtained by two glioma neuropathologists who performed independent immunohistochemical analyses of all of glioma samples. Tumor tissues included 12 cases of GBM, eight cases of AA, 10 cases of LGA, and six cases of OG. As normal controls, we used six non-tumoral white matter samples obtained from six anonymized patients (age range at the time of diagnosis, 21–54 years) undergoing focal brain resection during acute internal decompression operation for severe traumatic brain injury such as brain contusions [5, 32]. As normal controls, we choose white matter because tumor cells of gliomas originate from glial cells which are particularly abundant in white matter [17]. We obtained IRB approval prior to obtaining the normal white matter samples from the six patients [15]. Tumor and control tissues were frozen immediately after their removal at surgery and were kept at −70 °C until further use. The number of samples tested in each assay differed because of the limited amount of RNA that was available for each sample. The study obtained IRB approval regarding the use of human samples for experimental studies from the ethics committees of China–Japan Union Hospital and the First Hospital of Jilin University, China.

An astrocytoma cell line (SHG44) and two human GBM cell lines (U251 and BT325) were obtained from the Cell Center of the Chinese Academic Medical College (Beijing, China). Glioma cells were routinely cultured in Dulbecco’s modified Eagle’s medium (GIBCO®; Life Technologies, Inc., Gaithersburg, MD, USA) at 37 °C under 5 % CO_2_. Normal human astrocytes (NHA) (#CC-2565, Lot 80982; Lonza, Walkersville, MD, USA) were grown in a humidified incubator at 37 °C under 5 % CO_2_.

### RNA extraction and real-time RT-PCR

Total RNA was extracted from the clinical samples and cultured cells using Trizol (Invitrogen, Life Technologies, Carlsbad, CA, USA) according to the manufacturer’s instructions. Quantitative real-time PCR of *ADAR2* cDNA was performed using an Mx3000P real-time PCR instrument (Agilent, Santa Clara, CA, USA) with SYBR green I fluorescence, as follows. The reaction mixture consisted of 12.5 μL of 2× SYBR, 0.5 μL each of the forward (5′-GTATTTTGCCATGGA TATAGAAGATG-3′; sense, exon 1, 10 μmol L^−1^) and reverse primers (5′-GTACTGGGATCCAGGCTTGATCTCATTCAGCTG-3′; antisense, exon 2, 10 μmol L^−1^), and 2.0 μL of cDNA 2.0 μL, in a final reaction volume of 25 μL with DNase- and RNase-free water. Glyceraldehyde 3-phosphate dehydrogenase (*GAPDH*; assay ID Hs9999905_m1; Applied Biosystems, Foster City, CA, USA) was used as a reference gene. The PCR conditions consisted of 10 min at 95 °C, followed by 45 cycles of 95 °C for 20 s, 60 °C for 30 s, and 72 °C for 45 s. Real-time PCR was performed using serial dilutions of each cDNA template (1×, 1:4, 1:16, 1:64, and 1:256). The cycle threshold (Ct value) was defined as the number of cycles required to reach the fluorescence threshold. As a representative example, an amplification plot was generated for *ADAR2* in which fluorescence intensity was plotted against cycle number. The comparative Ct method was used to quantify transcript number, and the normalized Ct value (2^–ΔΔCt^) was used to estimate the level of *ADAR2* gene expression. The odds ratio and 95 % confidence interval were calculated automatically by UNPHASED software (MRC Biostatistics Unit, Cambridge, UK). Real-time assays were performed in triplicate from two independent RT reactions.

### RT-PCR

cDNA was synthesized using SuperScript II reverse transcriptase (Invitrogen) and random hexamer primers. To analyze human ADAR2 alternative splicing, we performed PCR reactions using forward (5′-GTATTTTGCCATGGATATAGAAGATG-3′; sense, exon 1) and reverse primers (5′-GTACTGGGATCCAGGCTTGATCTCATTCAGCTG-3′; antisense, exon 2), which amplified both splice variants from cDNA.

### Calculation of peritumoral brain edema (PTBE)

PTBE contributes to the morbidity and mortality of brain tumors. The development of PTBE is influenced by many factors. PTBE in brain tumors might be a prognostic indicator of patient survival [25]. The extent of PTBE and tumor size were measured by magnetic resonance imaging (MRI) [25]. The tumor volume was measured on enhanced T1 weighted images and the volume of edema surrounding the tumors was measured on T2 weighted images [33]. The edema/tumor volume ratio was defined as the edema index (EI), and was used as an indicator of PTBE [30]. EI was calculated as PTBE area/tumor area. Thus, if EI = 1, there is no PTBE [14]. An EI of ≤3 was defined as weak PTBE (+), an EI of >3 to <7 was defined as moderate PTBE (++), and an EI of ≥7 was defined as strong PTBE (+++) [33].

### Statistical analysis

SPSS for Windows (version 14.0; SPSS, Inc., Chicago, IL, USA) was used to perform the Mann–Whitney *U* test to examine the difference in ADAR2 expression between patients and controls. Survival rates were estimated by the Kaplan–Meier method and overall survival was compared between patients with (+) and without (–) an *ADAR2* ASV using the log-rank test. Statistical significance (*p* < 0.05) was determined by descriptive statistics.

## Results

### ADAR2 expression in human glioblastoma cell lines

**Fig. 1:**
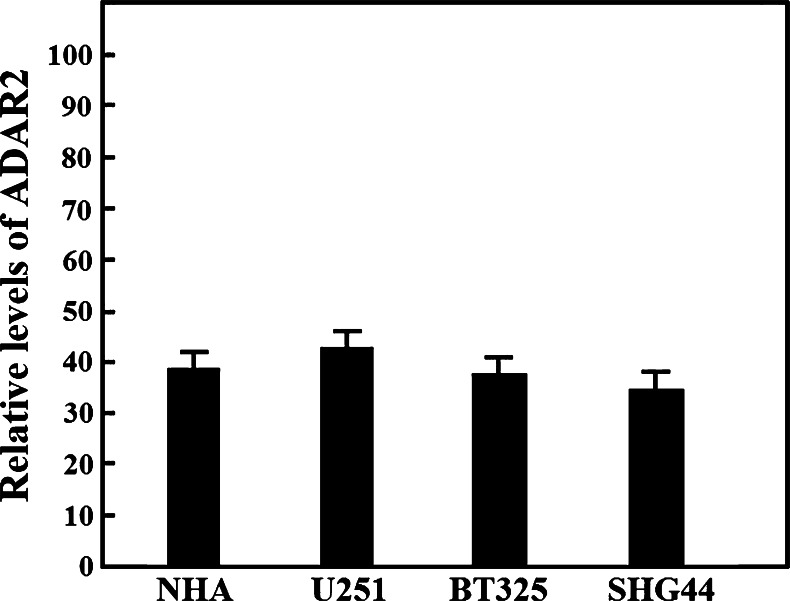
Relative *ADAR2* expression levels in human glioma cell lines. Real-time RT-PCR for *ADAR2* was performed using a human astrocytoma cell line (SHG44), human glioblastoma cell lines (U251 and BT325), and normal human astrocytes (NHA) as a control. *Error bars* indicate the standard deviation of triplicate experiments

Quantitative real-time RT-PCR revealed no marked differences in *ADAR2* mRNA expression between NHA, SHG44, U251, and BT325 cells (Fig. 1).

### ADAR2 mRNA expression in human gliomas

**Fig. 2:**
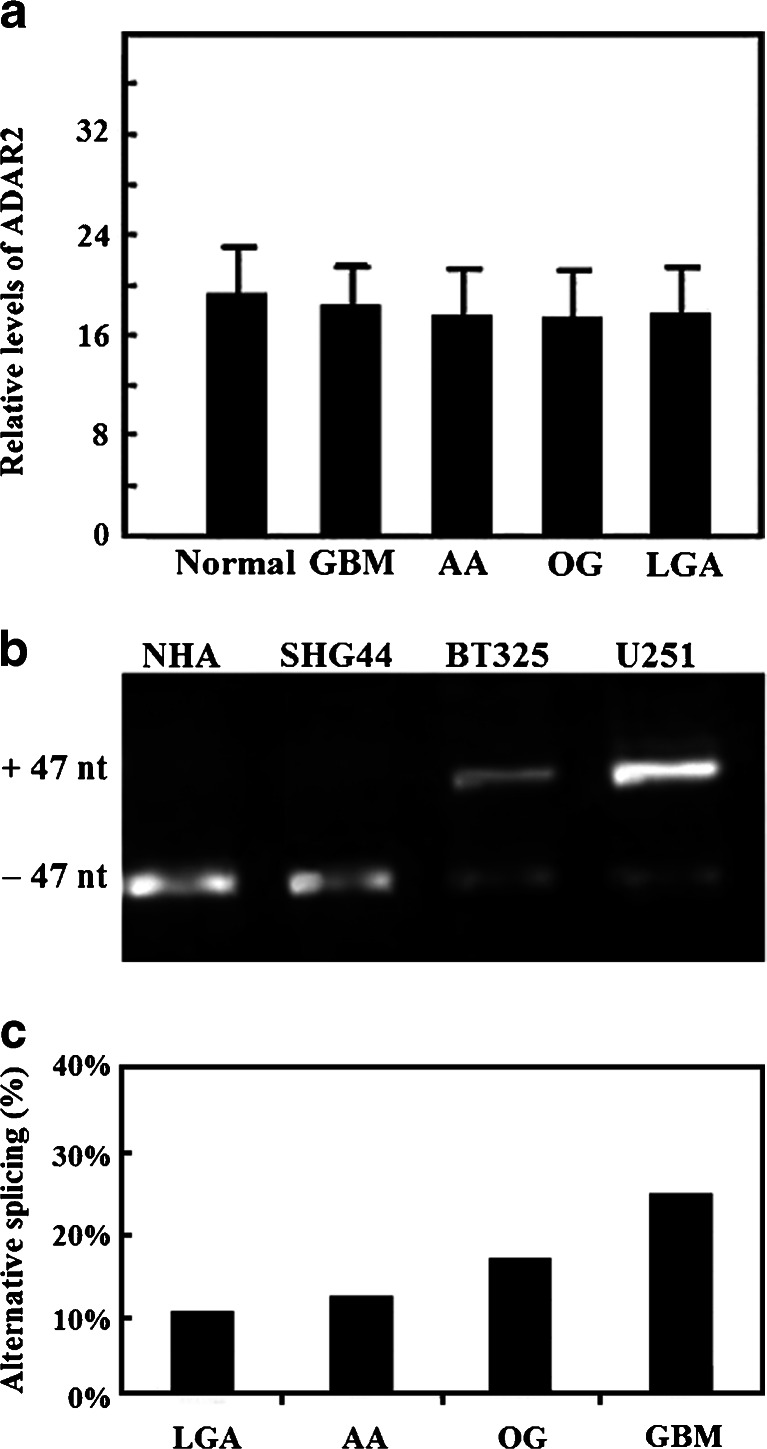
Expression of *ADAR2* mRNA in human glioblastomas and analysis of ADAR2 mRNA alternatively splicing variants (ASV). **a** Expression of *ADAR2* mRNA in human glioblastomas. Real-time PCR was performed to quantify the mRNA expression levels of *ADAR2* in normal tissues and in different grades and types of brain tumor. LGA, low-grade astrocytoma; AA, anaplastic astrocytoma; GBM, glioblastoma multiforme; OG, oligodendroglioma. Quantitative real-time PCR showed that the expression of *ADAR2* mRNA was not significantly different among the tumor tissues. **b** Analysis of *ADAR2* ASVs in glioma-derived cell lines and glioblastomas by RT-PCR. The migration position of the PCR reaction products corresponding to the transcripts with and without the 47-nucleotide insert are indicated. **c** Analysis of *ADAR2* ASVs in glioma tissues. The *ADAR2* ASV was detected in 1/10 LGAs, 1/6 OGs, 1/8 AAs, and 3/12 GBMs, corresponding to rate of self-editing-induced ASVs of 10 %, 16.7 %, 12.5 %, and 25 %, respectively

We performed semi-quantitative RT-PCR using 44 glioma samples, and performed quantitative real-time RT-PCR using six normal white matter tissue samples and 36 glioma samples (12 GBM, eight AA, 10 LGA, and six OG). We found that *ADAR2* mRNA expression was not significantly different between the gliomas and normal white matter tissues (all *p* > 0.05; Fig. 2a).

### Expression of ADAR2 ASVs in glioma cell lines

RT-PCR amplification of total RNA isolated from the NHA and SHG44 cells presented a single electrophoretic band. RT-PCR amplification of total RNA isolated from the glioma-derived cell line U251 and BT325 revealed two distinct electrophoretic bands (Fig. 2b). Sequence analysis of both isolated *ADAR2* cDNAs indicated that these two isoforms were produced by alternative splicing. The longer isoforms contained a 47-nucleotide sequence (GATCCTGCAACGAAGGCGTTGTAAGTTACTCTTTCTGGGCACCACAG) inserted after the first 28 nucleotides of the coding region. This ASV is identical to the one that was previously identified in a human *ADAR2* transcript containing a 47-nucleotide insertion at the analogous position [18, 20, 27].

### Expression of ADAR2 ASVs in glioma tissues

In glioma samples, we detected *ADAR2* ASV mRNA which was identical to that found in the U251 and BT325 cell lines. The ASV was not detected in any of the normal human brain tissue samples. The ADAR2 ASV was expressed in 1/10 LGAs, 1/6 OGs, 1/8 AAs, and 3/12 GBMs, corresponding to rates of self-editing induced ASVs of 10 %, 16.7 %, 12.5 %, and 25 %, respectively. The expression of the ASV was highest in GBMs, the most aggressive type of gliomas. The level of ASV expression was correlated with tumor grade (Fig. 2c).

### ADAR2 ASV expression is correlated with the extent of PTBE in patients with GBM

Patients with GBM were classified into two groups according to the presence (+; *n* = 3) or absence (–; *n* = 9) of the *ADAR2* ASV. For the three *ADAR2* ASV + patients, weak, moderate, and strong EI scores were observed in 0 (0 %), 0 (0 %), and three (100 %) patients, respectively. By contrast, the EI scores were classified as weak, moderate, and strong in seven (78 %), two (22 %), and 0 (0 %) in the *ADAR2* ASV – patients, respectively (Table 1). The EI scores were significantly greater in the *ADAR2* ASV + patients than in the *ADAR2* ASV – patients (*p* < 0.01).

**Table 1:** Correlation between *ADAR2* ASV expression and the edema/tumor volume ratio (EI)

	ADAR2 ASV (+)	ADAR2 ASV (-)	*P* value
	(n = 3)	(n = 9)	
EI			= 0.0005*
**−**	0 (0 %)	0 (0 %)	
**+**	0 (0 %)	7 (78 %)	
**+ +**	0 (0 %)	2 (22 %)	
**+ + +**	3 (100 %)	0 (0 %)	

A full description of the EI is described in “Materials and methods”. EI was graded as negative (–), weak (+), moderate (+ +), or strong (+ + +).

*Statistically significant by the Mann–Whitney *U* test

### ADAR2 ASV expression and the invasiveness of GBM

**Fig. 3:**
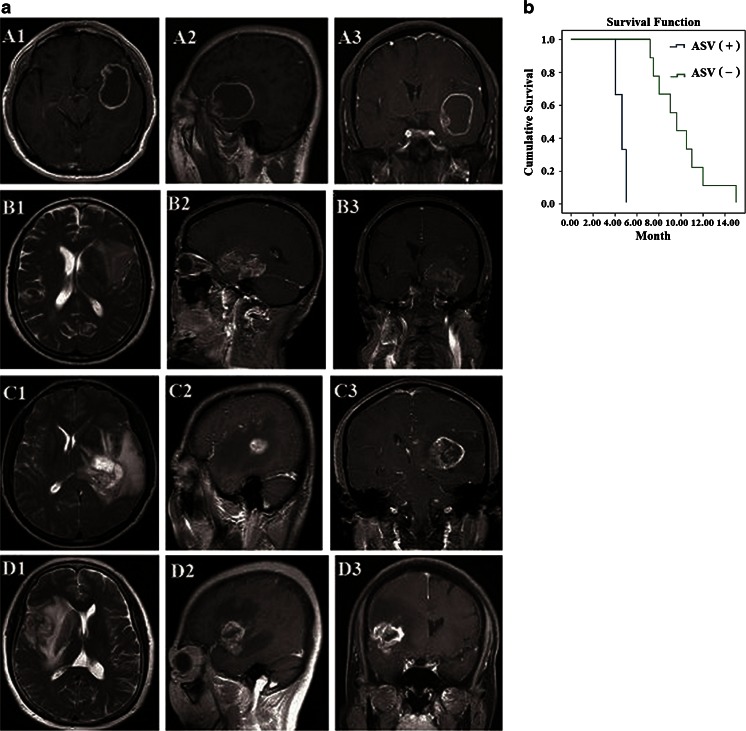
Magnetic resonance imaging (MRI) of glioblastomas and overall survival analysis of patients with GBM. **a** MRI of glioblastomas. *A* Patient with ADAR2 ASV– GBM. The lesion has invaded the temporal and frontal lobes. *B–D* Patients with ADAR2 ASV + GBM. *B* the lesion has invaded the temporal and frontal lobes. *C* The lesion has invaded the temporal, parietal, and occipital lobes. *D* The lesion has invaded the temporal, frontal, and parietal lobes. *A1* axial T1-weighted image; *A2, D2* sagittal T1-weighted images; *A3* coronal contrast-enhanced T1-weighted image; *B1*, *C1*, *D1* axial T2-weighted images; *B2*, *C2* sagittal contrast-enhanced T1-weighted image; *B3*, *C3*, *D3* coronal contrast-enhanced T1-weighted images. **b** Overall survival analysis of patients with GBM. The median survival time was significantly shorter in *ADAR2* ASV + patients compared with *ADAR2* ASV – patients (log-rank test, *p* < 0.0001)

To analyze the association between *ADAR2* ASV expression and invasiveness of GBM, we used MRI to examine the extent of tumor invasion in the GBM patients. For the three *ADAR2* ASV + patients, the tumor had invaded two lobes in one patient and three lobes in two patients. By contrast, among the nine *ADAR2* ASV – patients, the tumor had invaded one lobe in four patients, two lobes in four patients, and three lobes in one patient. Therefore, the tumors in the *ADAR2* ASV + patients were more aggressive than those in the *ADAR2* ASV – patients (*p* < 0.01; Fig. 3a).

### Overall survival in patients with GBM

The median survival time was 4.53 months for the three *ADAR2* ASV + patients compared with 9.98 months for the *ADAR2* ASV – patients. The difference in median survival time between the two groups of patients was statistically significant (*p* < 0.0001; Fig. 3b).

## Discussion

RNA editing is a common phenomenon in eukaryotic cells that leads to post-transcriptional base changes in mRNA. In mammals, a growing number of genes have been identified that undergo a type of RNA editing characterized by site-selective A-to-I modification [2, 3, 8]. The best-studied A-to-I editing substrates are the brain-specific transcripts coding the GluR. For GluR-B, the Q/R site controls the Ca^2+^ permeability of the ion channel [16, 34]. ADAR2 is the main enzyme responsible for recoding editing in the brain. It is predominantly expressed in neurons, and is weakly expressed in gliocytes [17]. Mammalian RNA editing catalyzed by ADAR1 and ADAR2 plays pivotal roles in the brain by controlling functional modifications of neurotransmitter receptors and ion channels [31]. The ADAR proteins and RNA editing targets have been studied in many human diseases associated with RNA editing, including various cancers [6, 7, 11, 12, 26, 28].

Gliomas are the most common malignant tumors in the central nervous system, and are almost always fatal. The molecular basis for malignant progression in gliomas involves several collaborative processes. Thus far, several studies have shown that a general state of underediting exists in gliomas in some RNA editing sites, including the Q/R site, the 5HT_2C_ serotonin receptor site, and the Glu/Arg site. For regulated normal cells, these findings suggest a key role of ADAR2 editing activity in controlling the growth of gliomas [5, 22, 26, 32] because this enzyme seems to prevent cell proliferation by the Akt pathway [16, 34] and/or modulates the cell cycle [10]. It was also reported that the Q/R site of GluR-B in gliomas is underedited compared with that in normal tissue. Underediting at this position might be due to decreased *ADAR2* expression or downregulation of ADAR2 enzymatic activity.

The recent findings regarding *ADAR2* mRNA expression in gliomas were somewhat inconsistent [5, 22, 26]. The first report, by Maas et al., showed that the *ADAR2* mRNA expression was not significantly altered in one OG and seven GBM samples, although the Q/R site of GluR-B was underedited compared with that in control tissues [22]. Paz et al. observed a prominent reduction in *ADAR2* mRNA levels in four types of brain tumors (GBM, LGA, AA, and OG) in an analysis of 18 brain tumors; however, their control was normal brain tissue containing neurons, which show high ADAR2 expression [26]. Cenci et al. analyzed 14 astrocytoma tissue samples from 10 children, and found no significant differences in *ADAR2* mRNA levels between tumor tissue and normal white matter [5].

In our study, we examined the expression of *ADAR2* mRNA in human glioblastoma cell lines and in NHA, and found no differences in its expression among these cell types. We also determined the expression of *ADAR2* mRNA in various gliomas of different grades, and found that it was not significantly different between gliomas and normal white matter. Based on the results of previous studies and our study, we consider that the expression of *ADAR2* mRNA is not significantly altered in gliomas. Thus, we speculate that underediting at the Q/R site in gliomas is not simply due to decreased *ADAR2* expression.

The human *ADAR2* gene comprises 14 exons [23, 29], and several ASVs have been identified [1, 18, 20, 23, 27, 29]. For example, one involving self-editing of *ADAR2* pre-mRNA creates a 3′-prime splice site within intron 1 leading to the insertion of a 47-nucleotide sequence into the intronic sequence. Another ASV includes an exon located 18 kb upstream of the previously annotated first coding exon, and extends the open reading frame of *ADAR2* by 49 amino acids [20]. In mammals, alternative splicing occurs within the catalytic domain, the RNA-binding domain, and at the carboxy terminus. Of these ASVs, some have no effect on editing activity whereas others decrease the editing activity of ADAR2. Therefore, the characterization of *ADAR2* transcription and alternative splicing is essential to understand the regulation of RNA editing.

The regulation of RNA editing activity in vivo remains unknown: the amount of total ADAR2 mRNA is one factor regulating RNA editing, but the level of total ADAR2 does not necessarily represent the editing activity, and alternative splicing may be another factor affecting RNA editing. Alternative splicing events have been demonstrated to affect the catalytic activity of ADAR2. Rueter et al. [27] demonstrated experimentally that the addition of 47 nucleotides to the 5’ end of exon2 occurs due to RNA editing within intron 1 and decreases ADAR2 activity in vivo because of the inefficient initiation of translation from Met25. The results of Rueter et al. are cited by several subsequent studies [18, 20]. In the present study, we identified an ASV containing a 47-nucleotide insertion by RT-PCR and sequencing. This ASV is identical to that identified by Rueter et al., which has lower RNA editing activity. We also detected that the expression rate of ASV is higher in glioma than in normal white matter. On this basis, we speculate that the expression of a less-active ASV of ADAR2 may correlate with downregulation of A-to-I editing in gliomas.

In our study, we found an *ADAR2* ASV in two glioma-derived cell lines, U251 and BT325, and in human glioma tissues. The ASV was detected in 10 %, 16.7 %, 12.5 %, and 25 % of LGA, OG, AA, and GBM tissues, respectively. The mRNA sequence of the *ADAR2* ASV was identical to that originally reported by Rueter et al. [27] and by many other authors since then. The splicing variant is generated via a proximal 3′ acceptor site, at which 47 nucleotides are added to the ADAR2 coding region, changing the reading frame of the mature ADAR2 transcript. Insertion of a 47-nucleotide cassette into the coding region shifts the translation start to an inefficient downstream methionine. In vitro and tissue culture model systems have indicated that the ASV downregulates the catalytic activity of the human ADAR2 protein isoforms without affecting the substrate. This finding is consistent with the assumption that downregulation of A-to-I editing in gliomas may be due to the expression of a less-active ASV of *ADAR2*.

We found that the positive rate of the self-editing-induced ASV increased significantly with increasing malignancy of the gliomas, being greatest in GBMs, the most malignant type of glioma. To determine whether *ADAR2* ASV expression was correlated with the malignant features of gliomas, we divided the patients with GBM into two groups according to expression of the ASV, and compared PTBE, invasiveness, and median survival time between the two groups. These analyses revealed that *ADAR2* ASV + patients had more severe PTBE, tumor invasion into more brain lobes, and had a shorter median survival time compared with *ADAR2* ASV – patients.

## Conclusions

Our data indicate that (1) ADAR2 mRNA levels were not altered in glioma-derived cell lines or in glioma tissues; (2) self-editing of ADAR2 pre-mRNA generates an ADAR2 ASV in glioma-derived cell lines and glioma tissues; and (3) expression of ADAR2 ASV may be correlated to the malignancy of gliomas. Identification of the mechanistic characterization of ADAR2 ASV may be helpful in the future development of individualized molecular-targeted therapy for glioma.

## Abbreviations

A-to-I: adenosine-to-inosine
AA: anaplastic astrocytomas
ADAR: adenosine deaminases acting on RNA
ASV: alternative splicing variant
Ct: cycle threshold
EI: edema index
GAPDH: glyceraldehyde 3-phosphate dehydrogenase
GBM: glioblastomas multiforme
GluR-B: glutamate receptor subunit B
LGA: low-grade astrocytomas
MRI: magnetic resonance imaging
NHA: normal human astrocytes
OG: oligodendrogliomas
PTBE: peritumoral brain edema
RT-PCR: reverse-transcription–polymerase chain reaction
